# Attitudinal predictors of older peoples’ and caregivers’ desire to deprescribe in hospital

**DOI:** 10.1186/s12877-019-1127-x

**Published:** 2019-04-15

**Authors:** Sion Scott, Allan Clark, Carol Farrow, Helen May, Martyn Patel, Michael J. Twigg, David J. Wright, Debi Bhattacharya

**Affiliations:** 1 School of Pharmacy, University of East Anglia, Norwich, Norfolk NR4 7TJ UK; 2 Pharmacy Department, Norfolk and Norwich University Hospitals NHS Foundation Trust, Colney Lane, Norwich, Norfolk NR4 7UY UK; 3 Norwich Medical School, University of East Anglia, Norwich, Norfolk NR4 7TJ UK; 4 Older People’s Medicine Department, Norfolk and Norwich University Hospitals NHS Foundation Trust, Colney Lane, Norwich, Norfolk NR4 7UY UK

**Keywords:** Potentially inappropriate medicines, Polypharmacy, Cessation, Questionnaire, Survey

## Abstract

**Background:**

Deprescribing is a partnership between practitioners, patients and caregivers. External characteristics including age, comorbidities and polypharmacy are poor predictors of attitude towards deprescribing. This hospital-based study aimed to describe the desire of patients and caregivers to be involved in medicine decision-making, and identify attitudinal predictors of desire to try stopping a medicine.

**Methods:**

Patients and caregivers recruited from seven Older People’s Medicine wards across two UK hospitals completed the revised Patients’Attitudes Towards Deprescribing (rPATD) questionnaire. Patients prescribed polypharmacy and caregivers involved in medication decision-making of such patients were eligible. A target of 150 patients and caregivers provided a 95% confidence interval of ±11.0% or smaller around rPATD item agreement. Descriptive statistics characterised participants and rPATD responses. Responses to items regarding desire to be involved in medication decision-making and desire to try stopping a medicine were used to address the aims. Binary logistic regression provided the adjusted odds ratios (OR) for predictors of desire to try stopping a medicine.

**Results:**

Patient participants (*N* = 75) were a median (IQ) 87.0 (83.0, 90.0) years old and the median (IQ) number of pre-admission medication was 8.0 (6.0, 10.0). Caregiver participants (*N* = 76) were a median (IQ) 70.0 (57.0, 83.0) years old and the majority were a spouse (63.2%). For patients and caregivers respectively, the following were reported: 58.7 and 65.8% wanted to be involved in medication decision-making; 29.3 and 43.5% reported a desire to try stopping a medicine. Attitudinal predictors of low desire to try stopping a medicine for patients and caregivers are a perception that there are no unnecessary prescribed medicines [OR = 0.179 (patients) and 0.044 (caregivers)] and no desire for dose reduction [OR = 0.199 (patients) and 0.024 (caregivers)]. A perception of not being prescribed too many medicines also predicted low patient desire to try stopping a medicine [OR = 0.195].

**Conclusion:**

A substantial proportion of patients and caregivers did not want to be involved medication decision-making, however this should not result in practitioners dismissing deprescribing opportunities. The three diagnostic indicators for establishing desire to try stopping a medicine are perceived necessity of the medicine, appropriateness of the number prescribed medications and a desire for dose reduction.

**Electronic supplementary material:**

The online version of this article (10.1186/s12877-019-1127-x) contains supplementary material, which is available to authorized users.

## Background

Potentially inappropriate medicines (PIMs) offer more risks than benefits and are associated with adverse outcomes including morbidity, hospitalisation and mortality [1]. A prospective study across six European hospitals reported between 34.7 and 77.3% of older people were prescribed a PIM on admission [2]. There is therefore a need to review medicines to determine suitability for discontinuation, a process termed ‘deprescribing’ [3]. While the prevalence of PIMs on hospital admission is high [2], deprescribing practice is limited and reactive in response to iatrogenic harm rather than proactive to prevent future harm [4]. Given that deprescribing requires an accurate medication history and monitoring to observe response to medication withdrawal [5], an admission to hospital where these two activities are routine, provides an opportunity to develop a deprescribing intervention.

Prescribing should be based on a partnership as the prescriber is the disease expert and the patient is the expert on their illness [6]. It is therefore unsurprising that patient engagement in decision-making is a proposed essential component of deprescribing [5]. Consultations between practitioners and patients are an opportunity to determine whether deprescribing is appropriate, agree strategies for ongoing monitoring and establish the patient’s desire to try deprescribing [5].

Trials across multiple settings report up to half of older patients decline deprescribing interventions [7–10]. Exploration of predictors for this lack of desire to deprescribe has focussed on external characteristics such as age, gender and number of prescribed medications. A recent retrospective analysis of hospital electronic medical records reported that all external characteristics analysed, including PIM prevalence, number of medicines at admission and comorbidities had no effect on patients’ willingness to accept deprescribing [10]. It is unsurprising that these characteristics do not predict desire to deprescribe as there is a substantial body of evidence in the field of behavioural science confirming that a key predictor of behaviour is attitude towards the behaviour, which is poorly predicted by external characteristics [11–13]. Furthermore, external demographic characteristics cannot be changed thus contribute little to guiding physicians or those developing deprescribing interventions. Identification of attitudinal predictors of desire to deprescribe may therefore provide modifiable targets for an intervention targeting patients’ and caregiver’ behaviour. Such attitudinal predictors are likely to be related to patients’ barriers and enablers to deprescribing, which have been characterised in a variety of settings [14]. Patient reported barriers include perceived continued need for medicines, fear of withdrawal effects and previous bad experiences. Conversely, experience or fear of side effects, absence of observable benefits and reduction in pill burden are patient reported enablers. The extent to which these potentially modifiable attitudinal factors predict desire to deprescribe remains unknown.

Informal caregivers such as family members are increasingly involved in medication decision-making. For patients that are unable to participate in these decisions, such as those living with cognitive impairment, caregivers frequently assume sole responsibility [15, 16]. Furthermore, caregivers influence engagement with deprescribing by physicians and patients who are able to participate in decision-making [14, 17]. Despite the wide ranging influence exerted by caregivers on the deprescribing processes, their level of engagement with and attitudinal factors influencing desire to deprescribe are unknown.

Older peoples’ attitudes towards deprescribing have been described using the Patients’ Attitudes Towards Deprescribing questionnaire (PATD) [18–21]. The majority of respondents in the reported studies indicate being satisfied with existing medication whilst incongruously, over 90% also indicate willingness to accept deprescribing proposed by a doctor. This high level of willingness contrasts the significant proportion of participants in deprescribing trials declining deprescribing propositions [7–10]. This gap between reported willingness and observed behaviour requires further exploration. Given that willingness to accept deprescribing proposed by a doctor has demonstrated limited variation in responses, this may not provide the best data for explaining this gap.

The revised Australian-validated Patients’ Attitudes Towards Deprescribing questionnaire (rPATD) explores factors that influence desire to deprescribe not captured by the PATD. The rPATD items are grouped into the four factors of *burden* of taking medication, *appropriateness* of medication (perceived harms and benefits), *concerns about stopping* the medication and level of *involvement* in making decisions about medicines. The *appropriateness* factor provides the new item “I would like to try stopping one of my medicines to see how I feel without it”. This item provides an indication of the patient’s attitude towards their prescribed medication by indicating their desire to try stopping a medicine. Furthermore, given that a significant proportion of previously reported deprescribing trials have been pharmacist-led [22], this item may provide better data for explaining the gap between reported willingness to accept deprescribing proposed by a doctor and observed declining of deprescribing propositions.

This hospital-based study aimed to describe the likely desire of patients and caregivers to be involved in medicine decision-making and, identify any attitudinal predictors of desire to try stopping a medicine.

## Methods

### Questionnaire refinement and testing

With the author’s consent, minor adaptations were made to the rPATD prior to UK use. People aged ≥65 years in the UK are exempt from prescription charges thus an item in the rPATD *burden* factor exploring views towards paying for medication was rephrased to explore perceptions of the National Health Service’s (NHS) medication expenditure. The item was phrased “I feel my medicines are value for money for the NHS” and appropriate variation for caregivers. The *burden* factor captures the burden, such as financial, on the individual patient (or caregiver), therefore this re-rephrased item relating to burden on the NHS no longer belongs to this factor. For the purposes of data reporting, the re-phrased question was presented separately from the *burden* factor under the heading *burden to the National Health Service*.

Face and content validity of the UK adapted rPATD were assessed using cognitive interviews with older people and caregivers. Cognitive interviewing allows evaluation of how the target audience perceives survey instruments and their constructs. This information is used to identify survey flaws and improve questions [23, 24]. Whilst there were no reasons to anticipate UK participants experiencing difficulties completing the Australian-validated rPATD, cognitive interviews were undertaken in light of the minor adaptation and to ensure face and content validity were retained in the UK context.

Cognitive interview participants self-completed the adapted rPATD with a researcher present and a concurrent ‘think-aloud’ with verbal probing identified and explored problems with interpreting and answering items [23, 24]. Participants were also asked whether any factors influencing attitudes towards deprescribing were underrepresented in the rPATD. Cognitive interviews continued until no further adaptations were deemed necessary as evidenced by participants reporting no problems with completing the rPATD.

### Administration of the UK adapted rPATD to patients and caregivers in hospital

#### Study sample and setting

Patients and visiting caregivers were independently recruited (i.e. they were not paired) from seven Older People’s Medicine (OPM) wards at one and two UK hospitals respectively. Criteria for patients triaged to an OPM ward were minimum age (ranging between 70 to 80 years across sites) and either multiple co-morbidities or physical frailty.

All inpatients from OPM wards prescribed at least five pre-admission medicines (polypharmacy [25]) were eligible. The number of pre-admission medicines was determined from the hospitals’ pharmacy medicines reconciliation records, which use at least two sources of information, such as a community pharmacy record and a patient’s own report, to establish an accurate medication history. Patients unable to speak or read English, deemed by the healthcare team as unable to provide informed consent, inappropriate to approach for recruitment for reasons such as being seriously unwell or unable to make informed decisions about medicines were excluded. For patients who were unable to provide informed consent or make informed decisions about medicines, any of their visitors during the study period were screened as caregiver participants. Accordingly, patients and caregivers were not paired.

All visitors self-reporting an unpaid role in managing the medication of an inpatient satisfying the inclusion criteria for the study’s patient participant arm were eligible as caregivers. Caregivers unable to speak or read English and aged < 18 years old were excluded.

#### Recruitment and survey administration

Patients were screened for eligibility and approached for inclusion by an OPM doctor, nurse or pharmacist. Patients expressing an interest were approached by a researcher who provided an information leaflet and answered questions. Written, informed consent was obtained for rPATD administration and collection of demographic information. The rPATD was self-completed on an electronic tablet by patients at the bedside with a researcher present to assist if necessary. Patient demographics and the number of pre-admission medicines were recorded.

Visitors of OPM wards were screened by a research nurse for eligibility to determine whether they were caregivers self-reporting an unpaid role in managing the medication of an OPM patient. Only one caregiver per OPM patient was approached for recruitment as it was deemed unethical by the study team’s patient and caregiver members to approach several caregivers for one patient. As no identifiable personal information was collected from caregivers, consent was implied through self-completion of the questionnaire. Caregivers who agreed to participant were provided with a questionnaire pack including an anonymous demographic information collection form and the rPATD. Caregivers were invited to self-complete the questionnaire and provide demographic information for themselves and their care recipient in addition to indicating their relationship with the care recipient and the number of pre-admission medications. Caregivers were instructed to return the pack to a member of ward staff.

Participants were asked to respond to the rPATD reflecting on medication as prescribed prior to admission (pre-admission medicines) but in the context of deprescribing in the hospital setting.

#### Sample size

No participant data are reported for the rPATD to inform sample size estimation. Participant data from the original PATD indicate a maximum distribution across the response items of 65 to 35%, representing the ‘worst case scenario’ in terms of precision [20]. This was reported for the item “I feel that I am taking a large number of medications”. Accordingly, response for all other items are estimated to a greater degree of precision. Based on the 65 to 35% PATD response distribution, assuming a similar distribution for the rPATD and anticipated minimal adaptations required for UK use, a sample of 75 participants per population provides a 95% confidence interval (CI) of ±11.0% or smaller around the estimates of agreement with each rPATD item. This sample size is therefore appropriate for ensuring that there is no overlap in CIs between the proportion of respondents agreeing and disagreeing with an rPATD item.

#### Statistical analysis

Analyses were performed using IBM SPSS Statistics version 23.0 for Windows. Descriptive statistics were used to characterise the participants and rPATD responses. Items are reported grouped under the four rPATD factors; *burden*, *appropriateness*, *concerns about stopping* and *involvement*. Global item 1 captures willingness to accept deprescribing proposed by a doctor and global item 2 captures satisfaction with current medications.

The primary outcome of desire to try stopping a medicine was the *appropriateness* question “I would like to try stopping one of my medicines to see how I feel without it” (patients) and “I would like the doctor to try stopping one of my care recipient’s medicines to see how they feel without it” (caregivers).

In order to identify respondents with a desire to try stopping a medicine, responses to the primary outcome, *involvement* item relating to likely desire to be involved in medicine decision-making and the two global rPATD questions were dichotomised into those in agreement (agree and strongly agree) and those ambivalent or in disagreement (strongly disagree, disagree and neither agree nor disagree).

Backward binary logistic regression was performed between statements in the four factors and the primary outcome. To identify perceived barriers predicting desire to try stopping a medicine, responses to each statement were dichotomised into those who disagreed that it was a barrier (strongly disagree and disagree) and those who were ambivalent or in agreement (neither agree nor disagree, agree and strongly agree) that it was a barrier. Variables with less than 5.0% distribution in responses cross-tabulated with the primary outcome were excluded as it was felt that these had insufficient variability to be reliability modelled.

## Results

### Questionnaire testing and refinement

After three cognitive interviews with patients and caregivers, no further recommendations for improving the rPATD items were suggested. Patient participant’s ages ranged between 69 and 73 years and two were male. Patients were taking between five and 15 medicines. All three caregiver participants were female aged between 28 and 54 years and, two of their care recipients were female. Caregivers’ care recipients were taking between five and 11 medicines.

No recommendations for improving the original rPATD items were identified. However, the first participant, a caregiver, expressed difficulty with responding to the adapted item regarding NHS spending on medication, citing insufficient knowledge of the cost-effectiveness of medicines. This in turn led to difficulties with expressing a view on whether they felt medicines were providing value for money to the NHS. The participant acknowledged the relevance of exploring views towards medication expenditure and suggested rephrasing the item as follows: “I feel the NHS spends a lot of money on my care recipient’s medicines”. The proposed revision was accepted by the research team and presented to subsequent participants, with appropriate adaptation for the patient rPATD version. The adapted item was acceptable to the remaining two caregivers and three patients, thus no further refinements were necessary. No additional factors potentially influencing attitudes towards deprescribing not already present in the rPATD were proposed. As face and content validity were demonstrated, no further adaptations to the rPATD were necessary.

### Administration of the adapted rPATD to patients and caregivers in hospital

**Fig. 1:**
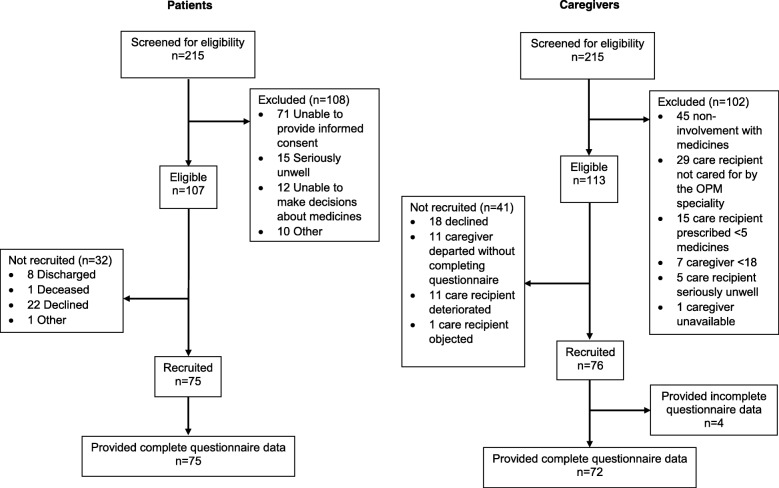
Participant flow

Figure 1 summarises recruitment of patients and caregivers; the primary reason for patient ineligibility was being unable to provide informed consent. For caregivers, non-involvement with medicines was the primary reason for exclusion.

### Patients

Recruitment of the target 75 patients from those eligible produced a recruitment rate of 70.1% (95% CI: 52.7, 87.5). Patient participant demographics are provided in Table 1.

**Table 1:** Patient, caregiver and care recipient demographics

Patient participants	
Median (IQ) age	87.0 (83.0, 90.0)
Number (%) female	34 (45.3)
Median (IQ) number of pre-admission medicines	8.0 (6.0, 10.0)
Caregiver participants	
Relationship to care recipient	
Number (%) spouse or partner	35 (46.1)
Number (%) non-spouse or partner relative	41 (53.9)
Median (IQ) caregiver age	70.0 (57.0, 83.0)
Median (IQ) care recipient age	86 (83.0, 89.0)
Number (%) female caregivers	47 (61.8)
Number (%) female caregivers	48 (63.2)
Median (IQ) number of pre-admission medicines prescribed to care recipient	8.0 (6.0, 10.3)

### Responses to the rPATD questionnaire

Table 2 illustrates patients’ rPATD responses. Agreement with deprescribing proposed by a doctor was high, with 97.4% (95% CI 93.8–100.0) agreeing with global item 1 (If my doctor said it was possible I would be willing to stop one or more of my regular medicines). Conversely, only 29.3% (95% CI 19.0–39.6) agreed with the primary outcome item (I would like to try stopping one of my medicines to see how I feel without it). A further 92.0% (95% CI 85.9–98.1) agreed with global item 2 (Overall, I am satisfied with my current medicines), indicating high satisfaction with current medications. Just over half (58.7% (95% CI 47.6–69.8)) of patients expressed a desire to be involved in medication-decision making in response to the relevant *involvement* item (I like to be involved in making decisions about my medicines with my doctors).

**Table 2:** Patients’ responses to the rPATD questionnaire

Item	Strongly disagree Number (%)	Disagree Number (%)	Neither agree nor disagree Number (%)	Agree Number (%)	Strongly agree Number (%)
Burden to the National Health Service					
I feel the National Health Service (NHS) spends a lot of money on my medicines	1 (1.3)	4 (5.3)	7 (9.3)	44 (58.7)	19 (25.3)
Burden					
Taking my medicines every day is very inconvenient	22 (29.3)	37 (49.3)	4 (5.3)	11 (14.7)	1 (1.3)
I feel that I am taking a large number of medicines	6 (8.0)	26 (34.7)	7 (9.3)	27 (36.0)	9 (12.0)
I feel that my medicines are a burden to me	21 (28.0)	38 (50.7)	5 (6.7)	7 (9.3)	4 (5.3)
Sometimes I think I take too many medicines	11 (14.7)	26 (34.7)	7 (9.3)	23 (30.7)	8 (10.7)
Appropriateness					
I feel that I may be taking one or more medicines that I no longer need	13 (17.3)	29 (38.7)	8 (10.7)	17 (22.7)	8 (10.7)
I would like to try stopping one of my medicines to see how I feel without it^a^	14 (18.7)	30 (40.0)	9 (12.0)	16 (21.3)	6 (8.0)
I would like my doctor to reduce the dose of one or more of my medicines	13 (17.3)	32 (42.7)	15 (20.0)	11 (14.7)	4 (5.3)
I think one or more of my medicines may not be working	12 (16.0)	26 (34.7)	22 (29.3)	13 (17.3)	2 (2.7)
I believe one or more of my medicines may be currently giving me side effects	21 (28.0)	29 (38.7)	4 (5.3)	15 (20.0)	6 (8.0)
Concerns about stopping					
I would be reluctant to stop a medicine that I had been taking for a long time	7 (9.3)	35 (46.7)	5 (6.7)	21 (28.0)	7 (9.3)
If one of my medicines was stopped, I would be worried about missing out on future benefits	14 (18.7)	28 (37.3)	5 (6.7)	25 (33.3)	3 (4.0)
I get stressed whenever changes are made to my medicines	17 (22.7)	39 (52.0)	7 (9.3)	10 (13.3)	2 (2.7)
If my doctor recommended stopping a medicine, I would feel that he/she was giving up on me	30 (40.0)	31 (41.3)	2 (2.7)	8 (10.7)	4 (5.3)
I have had a bad experience when stopping a medicine before	53 (70.7)	11 (14.6)	5 (6.7)	4 (5.3)	2 (2.7)
Involvement					
I have a good understanding of the reasons I was prescribed each of my medicines	6 (8.0)	3 (4.0)	6 (8.0)	38 (50.7)	22 (29.3)
I know exactly what medicines I am currently taking, and/or I keep an up-to-date list of my medicines	5 (6.7)	10 (13.3)	3 (4.0)	28 (37.3)	29 (38.7)
I like to know as much as possible about my medicines	3 (4.0)	10 (13.3)	5 (6.7)	35 (46.7)	22 (29.3)
I like to be involved in making decisions about my medicines with my doctors	6 (8.0)	19 (25.3)	6 (8.0)	23 (30.7)	21 (28.0)
I always ask my doctor, pharmacist or other healthcare professional if there is something I don’t understand about my medicines	1 (1.3)	13 (17.3)	2 (2.7)	33 (44.0)	26 (34.7)
Global					
If my doctor said it was possible I would be willing to stop one or more of my regular medicines	1 (1.3)	1 (1.3)	0	50 (66.7)	23 (30.7)
Overall, I am satisfied with my current medicines	0	1 (1.3)	5 (6.7)	49 (65.3)	20 (26.7)

^a^Primary outcome item

### Regression analysis

Items from all four factors were entered into the regression analysis. The resulting model predicted 62.9% (Negelkerke R^2^) of the variance and the Hosmer and Lemeshow goodness-of-fit test implied the model’s estimates fit the data to an acceptable level (*p* = 0.238). rPATD items predicting patients’ lack desire to try stopping a medicine are provided in Table 3. The full patient regression analysis is provided in Additional file 1: Table S1.

**Table 3:** rPATD items predicting patients’ and caregivers’ lack of desire to try stopping a medicine

Predictor rPATD item	Unadjusted odds ratio (95% confidence interval)	*p*-value	Adjusted odds ratio (95% confidence interval)	*p*-value
Patients				
Sometimes I think I take too many medicines	0.072 (0.023–0.231)	< 0.001	0.195 (0.045–0.841)	0.028
I feel that I may be taking one or more medicines that I no longer need	0.075 (0.025–0.229)	< 0.001	0.179 (0.044–0.726)	0.016
I would like my doctor to reduce the dose of one or more of my medicines	0.066 (0.021–0.206)	< 0.001	0.199 (0.050–0.787)	0.021
Caregivers				
I feel that the person that I care for may be taking one or more medicines that they no longer need	0.092 (0.030–0.279)	< 0.001	0.044 (0.006–0.310)	0.002
I would like the doctor to reduce the dose of one or more of my care recipient’s medicines	0.025 (0.007–0.094)	< 0.001	0.024 (0.004–0.137)	< 0.001

### Caregivers

The caregiver arm over recruited by one participant producing a recruitment rate of 67.2% (95% CI: 49.9, 84.5) for the 76 caregivers who completed the questionnaire. Caregiver and care recipient demographics are provided in Table 1.

### Responses to the rPATD questionnaire

Table 4 illustrates caregivers’ rPATD responses. Agreement with deprescribing proposed by a doctor was high, with 76.3% (95% CI 66.7–85.9) of caregivers agreeing with global item 1 (If their doctor said it was possible I would be willing to stop one or more of my care recipient’s medicines). Conversely, only 43.5% (95% CI 32.4–54.6) agreed with the primary outcome (I would like the doctor to try stopping one of my care recipient’s medicines to see how they feel without it). A further 80.3% (95% CI 71.3–89.3) agreed with global item 2 (Overall, I am satisfied with my care recipient’s current medicines), indicating high satisfaction with current medications. Approximately two thirds of caregivers (65.8% (95% CI 55.1–76.5)) expressed a desire to be involved in medication-decision making in response to the relevant *involvement* item (I like to be involved in making decisions about my care recipients medicines with their doctors).

**Table 4:** Caregivers’ responses to the rPATD questionnaire

Item	Strongly disagree Number %	Disagree Number %	Neither agree nor disagree Number %	Agree Number %	Strongly agree Number %
Burden to the National Health Service					
I feel the National Health Service (NHS) spends a lot of money on my care recipient’s medicines	0	5 (6.6)	28 (36.8)	26 (34.2)	17 (22.4)
Burden					
I feel that the person I care for is taking a large number of medicines	4 (5.3)	14 (18.4)	18 (23.7)	31 (40.8)	9 (11.8)
I feel that my care recipient’s medicines are a burden to them	8 (10.5)	37 (48.7)	14 (18.4)	16 (21.1)	1 (1.3)
Sometimes I think the person I care for takes too many medicines	8 (10.5)	21 (27.6)	23 (30.3)	21 (27.6)	3 (3.9)
Appropriateness					
I feel that the person that I care for may be taking one or more medicines that they no longer need	4 (5.3)	22 (28.9)	22 (28.9)	25 (32.9)	3 (3.9)
I would like the doctor to try stopping one of my care recipient’s medicines to see how they feel without it^a^	7 (9.2)	22 (28.9)	14 (18.4)	29 (38.2)	4 (5.3)
I would like the doctor to reduce the dose of one or more of my care recipient’s medicines	7 (9.2)	22 (28.9)	29 (38.2)	16 (21.1)	2 (2.6)
I think one or more of my care recipient’s medicines may not be working	5 (6.6)	22 (28.9)	32 (42.1)	17 (22.4)	0
I believe one or more of my care recipient’s medicines may be currently giving them side effects	6 (7.9)	25 (32.9)	18 (23.7)	23 (30.3)	4 (5.3)
Concerns about stopping					
I would be reluctant to stop one of my care recipient’s medicines that they had been taking for a long time	2 (2.6)	20 (26.3)	13 (17.1)	35 (46.1)	6 (7.9)
I get stressed whenever changes are made to my care recipient’s medicines	16 (21.1)	28 (36.8)	19 (25.0)	13 (17.1)	0
I feel that if I agreed to stopping one of my care recipient’s medicines then this is giving up on them	15 (19.7)	29 (38.2)	16 (21.1)	13 (17.1)	3 (3.9)
The person that I care for has had a bad experience when stopping a medicine before	43 (56.6)	22 (28.9)	6 (7.9)	5 (6.6)	0
Involvement					
I know exactly what medicines the person that I care for is currently taking and/or I have an up-to-date list of their medicines	0	12 (15.8)	3 (3.9)	41 (53.9)	16 (21.1)
I like to know as much as possible about my care recipient’s medicines	0	3 (3.9)	8 (10.5)	39 (51.3)	22 (28.9)
I like to be involved in making decisions about my care recipients medicines with their doctors	2 (2.6)	10 (13.2)	10 (13.2)	33 (43.4)	17 (22.4)
I always ask the doctor, pharmacist or other healthcare professional if there is something I don’t understand about my care recipient’s medicines	1 (1.3)	10 (13.2)	7 (9.2)	39 (51.3)	15 (19.7)
Global					
If their doctor said it was possible I would be willing to stop one or more of my care recipient’s medicines	2 (2.6)	1 (1.3)	14 (18.4)	50 (65.8)	8 (10.5)
Overall, I am satisfied with my care recipient’s current medicines	2 (2.6)	2 (2.6)	10 (13.2)	51 (67.1)	10 (13.2)

^a^Primary outcome item

### Regression analysis

Item 1 from *burden* and 2 from *involvement* were not entered into the regression due to insufficient distribution across responses. All remaining questions across the factors were entered into the binary logistic regression analysis with the primary outcome. The resulting model predicted 70.1% of the variance (Negelkerke R^2^) and the Hosmer and Lemeshow goodness-of-fit test implied the model’s estimates fit the data to an acceptable level (*p* = 0.852). rPATD items predicting caregivers’ lack of desire to try stopping a medicine are provided in Table 3. The full caregiver regression analysis is provided in Additional file 2: Table S2.

## Discussion

Engagement of patients and caregivers is a core component of deprescribing, yet a substantial proportion indicated limited desire to be involved in medication decision-making. Furthermore, the low desire to try stopping a medicine is in agreement with the substantial proportions of participants declining deprescribing in the trial environment [7–10]. However, patients and caregivers overwhelmingly report agreement with deprescribing proposed by a doctor. Practitioners should not therefore dismiss deprescribing opportunities due to patients and caregivers choosing to be less involved in decision-making. The three diagnostic indicators for establishing desire to try stopping a medicine are perceptions of the number and necessity of medicines and, a desire for dose reduction. These may also assist physicians with targeting relevant attitudinal predictors during deprescribing discussions.

Given similarities between the two English-speaking nations, minimal adaptations to the Australian rPATD were required before UK use. The item exploring burden of paying for medication was adapted to reflect the UK context. Whilst the sample size estimation was based on PATD data, the observed variation in responses to the global items was comparable, yielding confidence intervals equal to or narrower than predicted. The high consent rates afford some confidence in the generalisability of the findings to the populations of the hospitals at which the research was conducted. The presence of a researcher to support patients self-completing the rPATD may have biased responses. However, similarities with the caregiver rPATD responses indicate that researcher presence is unlikely to have unduly influenced the findings.

Half of potentially eligible patients were excluded due to inability to participate in medication decision-making. Inclusion of caregivers therefore provides representation of this previously under-researched population [18–20, 26]. The patient participant population is comparable to previous PATD studies [18–20, 26] and to a pan European study evaluating older people’s hospital admissions [2]. The caregiver population was comparable with a US study exploring treatment preferences of caregivers involved in medication decision-making [15]. These similarities indicate that the study findings may be generalisable beyond the two hospital study sites.

Similar to previous patient PATD responses in the outpatient clinic, acute hospital and care home settings, the global rPATD items in the present study demonstrated little variation, with the majority of respondents agreeing with deprescribing proposed by a doctor whilst also being satisfied with current medicines [18–20, 26]. There was, however, greater variation in responses to the items relating to patients’ and caregivers’ desire to be involved in medicine decision-making. This agrees with the existing literature in relation to some older people expressing preference for a passive role in decision-making [27–31] and may also be true of caregivers, who were similarly older in age [32].

Medication expenditure was acknowledged as a burden to the NHS by the majority of respondents, however this did not predict desire to try stopping a medicine. Patients did not consider their medications a burden as evidenced by no items in the *burden* factor attracting general agreement. Caregiver responses were similar, however the majority felt care recipients were taking a large number of medicines.

The *appropriateness* factor demonstrated greatest divergence between patients and caregivers. The majority of patients perceived their medicines were appropriate, whereas caregivers were ambivalent. This may be due to caregivers feeling that they lack understanding of their care recipient’s treatments [27].

Whilst there is qualitative literature indicating that deprescribing generates concerns for patients [17], the majority of patient respondents indicated that they did not hold concerns about stopping medication. This may be due to differences between actively inviting people to generate potential concerns versus inviting an opinion on specific concerns as in the present study [33]. Caregiver responses were similar to patients’, however resistance to deprescribing long-standing medication was conveyed but did not predict lack desire to try stopping a medicine. Physicians report reluctance to propose deprescribing for fear of patients perceiving this as withdrawal of care [14]; the present study suggests neither patients nor caregivers hold this view.

Some caution should be applied to this message, as whilst the majority of respondents agreed with deprescribing proposed by a doctor, they also reported content with existing medication. This potentially reflects a desire to conform, which may lead to agreement with a doctor’s recommendation to deprescribe despite concerns [31] and reluctance to report adverse outcomes such as return of symptoms [30].

The reported preference for a passive role in medication decision-making by older people in the literature [27] was expressed by some patients and caregivers in their responses to items in the *involvement* factor. Whilst items relating to the passive behaviour of knowledge acquisition regarding prescribed medicines attracted high agreement, the item relating to liking to be involved in decisions about medicines was lower.

The attitudinal predictors of desire to try stopping a medicine for both patients and caregivers are perceived necessity and a desire for dose reduction. As both items are from the *appropriateness* factor, this may represent a limitation of using an *appropriateness* item as the primary outcome. However, this could also suggest that attitude towards the *appropriateness* of medication is the most suitable target for a behaviour change intervention. Additionally, the predictive ability of the *burden* item regarding taking too many medicines for patients and not for caregivers suggests that a patients’ perceived burden of medicine taking is an important indicator of their desire to try stopping a medicine.

As the target behaviour is deprescribing and a key predictor of deprescribing is attitude towards deprescribing [11–13], the three attitudinal predictors are potential intervention targets. The finding that perceived medication necessity and a desire for dose reduction are predictors of both patients’ and caregivers’ desire to try stopping a medicine may offer efficiencies for intervention design. Behaviour change techniques offer an evidence-based approach to modifying attitudes towards a behaviour. For example, a practitioner may identify that a patient is prescribed an inappropriate medicine who is ambivalent to deprescribing. The present study indicates that one or more of three attitudinal predictors of desire to try stopping a medicine may alter this ambivalence. For example, the patient’s perception that they are not taking too many medicines can be targeted with the evidence-based behaviour change technique ‘information about emotional consequences’ [34, 35]. This theory-based approach to changing patients’ attitude towards deprescribing has been reported in the EMPOWER trial, which includes the behaviour change technique ‘information about health consequences’ [36].

## Conclusions

Patients and caregivers overwhelmingly report agreement with deprescribing proposed by a doctor yet vary in the extent to which they want to be involved in medicine decision-making. Practitioners should not therefore dismiss deprescribing opportunities due to patients and caregivers choosing to be less involved in decision-making. Three attitudinal predictors of reported desire to try stopping a medicine provide modifiable targets for developing a hospital deprescribing intervention targeting patients’ and caregivers’ behaviour. Future work should focus on identifying and testing evidence based behaviour change techniques targeting these attitudinal predictors for inclusion in deprescribing interventions.

## Additional files

Additional file 1: **Table S1.** Patient binary logistic regression model**.** Full binary logistic regression model data. (DOCX 16 kb)

Additional file 2: **Table S2**. Caregiver binary logistic regression model. Full binary logistic regression model data. (DOCX 16 kb)

## Abbreviations

CI: Confidence interval
IQ: Interquartile
NHS: National Health Service
OPM: Older People’s Medicine
OR: Odds ratio
PATD: Patients’ Attitudes Towards Deprescribing questionnaire
PIMs: Potentially inappropriate medicines
rPATD: Revised Patients’Attitudes Towards Deprescribing
