# Supplementary material for: Attitudinal predictors of older peoples’ and caregivers’ desire to deprescribe in hospital
Source: BMC Geriatr. 2019 Apr 15;19:108. doi: 10.1186/s12877-019-1127-x (PMC6466740; doi:10.1186/s12877-019-1127-x)
Supplement: Supplementary file 1 — Table S1. Patient binary logistic regression model. Full binary logistic regression model data. (DOCX 16 kb) [file 12877_2019_1127_MOESM1_ESM.docx]

**Additional table 1** Patient binary logistic regression model

| **Question** | | **No. % ‘yes’ disagreeing with outcome** | **No. % ‘no’ disagreeing with outcome** | **Unadjusted OR** | **p-value** | **Adjusted OR** | **p-value** |
| --- | --- | --- | --- | --- | --- | --- | --- |
| *Burden* | | | | | | | |
| 1 | I feel the National Health Service (NHS) spends a lot of money on my medicines | 41 (58.6) | 3 (60.0) | 0.943 | 0.950 |  |  |
| 2 | Taking my medicines every day is very inconvenient | 7 (43.8) | 37 (62.7) | 0.462 | 0.177 |  |  |
| 3 | I feel that I am taking a large number of medicines | 20 (46.5) | 24 (46.5) | 0.290 | 0.015* |  |  |
| 4 | I feel that my medicines are a burden to me | 5 (31.3) | 39 (66.1) | 0.233 | 0.016* |  |  |
| **5** | **Sometimes I think I take too many medicines** | **12 (31.6)** | **32 (86.5)** | **0.072** | **<0.001**** | **0.195** | **0.045*** |
| *Appropriateness* | | | | | | | |
| **1** | **I feel that I may be taking one or more medicines that I no longer need** | **9 (27.3)** | **35 (83.3)** | **0.075** | **<0.001**** | **0.179** | **0.016*** |
| 2 | I would like to try stopping one of my medicines to see how I feel without it (entered as the dependent variable/primary outcome) |  |  |  |  |  |  |
| **3** | **I would like my doctor to reduce the dose of one or more of my medicines** | **7 (23.3)** | **37 (82.2)** | **0.066** | **<0.001**** | **0.199** | **0.021*** |
| 4 | I think one or more of my medicines may not be working | 14 (37.8) | 30 (78.9) | 0.162 | 0.001* |  |  |
| 5 | I believe one or more of my medicines may be currently giving me side effects | 11 (44.0) | 33 (66.0) | 0.405 | 0.071 |  |  |
| *Concerns about stopping* | | | | | | | |
| 1 | I would be reluctant to stop a medicine that I had been taking for a long time | 19 (57.6) | 25 (59.5) | 1.084 | 0.865 |  |  |
| 2 | If one of my medicines was stopped, I would be worried about missing out on future benefits | 20 (60.6) | 24 (57.1) | 0.867 | 0.762 |  |  |
| 3 | I get stressed whenever changes are made to my medicines | 9 (47.4) | 35 (35) | 1.852 | 0.250 |  |  |
| 4 | If my doctor recommended stopping a medicine, I would feel that he/she was giving up on me | 8 (57.1) | 36 (59.0) | 1.080 | 0.898 |  |  |
| 5 | I have had a bad experience when stopping a medicine before | 7 (63.6) | 37 (57.8) | 0.783 | 0.718 |  |  |
| *Involvement* | | | | | | | |
| 1 | I have a good understanding of the reasons I was prescribed each of my medicines | 43 (65.2) | 1 (11.1) | 14.957 | 0.013* |  |  |
| 2 | I know exactly what medicines I am currently taking, and/or I keep an up-to-date list of my medicines | 38 (63.3) | 6 (40.0) | 2.591 | 0.107 |  |  |
| 3 | I like to know as much as possible about my medicines | 36 (58.1) | 8 (61.5) | 0.865 | 0.817 |  |  |
| 4 | I like to be involved in making decisions about my medicines with my doctors | 29 (58.0) | 15 (60.0) | 0.921 | 0.868 |  |  |
| 5 | I always ask my doctor, pharmacist or other healthcare professional if there is something I don’t understand about my medicines | 35 (57.4) | 9 (64.3) | 0.748 | 0.637 |  |  |

Concerns factor reversed (no=1, yes=0)

Outcome reversed (no=1, yes=0)
