# Supplementary material for: Attitudinal predictors of older peoples’ and caregivers’ desire to deprescribe in hospital
Source: BMC Geriatr. 2019 Apr 15;19:108. doi: 10.1186/s12877-019-1127-x (PMC6466740; doi:10.1186/s12877-019-1127-x)
Supplement: Supplementary file 2 — Table S2. Caregiver binary logistic regression model. Full binary logistic regression model data. (DOCX 16 kb) [file 12877_2019_1127_MOESM2_ESM.docx]

**Additional table 2** Caregiver binary logistic regression model

| **Question** | | **No. % ‘yes’ disagreeing with outcome** | **No. % ‘no’ disagreeing with outcome** | **Unadjusted OR** | **p-value** | **Adjusted OR** | **p-value** |
| --- | --- | --- | --- | --- | --- | --- | --- |
| *Burden* | | | | | | | |
| 1 | I feel the National Health Service (NHS) spends a lot of money on my care recipient’s medicines | 25 (35.2) | 4 (80.4) | 0.136 | 0.081 |  |  |
| 2 | I feel that the person I care for is taking a large number of medicines | 20 (34.5) | 9 (50.0) | 0.526 | 0.240 |  |  |
| 3 | I feel that my care recipient’s medicines are a burden to them | 7 (22.6) | 22 (48.9) | 0.305 | 0.023* |  |  |
| 4 | Sometimes I think the person I care for takes too many medicines | 10 (21.3) | 19 (65.5) | 0.142 | <0.001*** |  |  |
| *Appropriateness* | | | | | | | |
| **1** | **I feel that the person that I care for may be taking one or more medicines that they no longer need** | **5 (10.6)** | **24 (82.8)** | **0.092** | **<0.001***** | **0.056** | **0.005** |
| 2 | I would like the doctor to try stopping one of my care recipient’s medicines to see how they feel without it (entered as the dependent variable/primary outcome) |  |  |  |  |  |  |
| **3** | **I would like the doctor to reduce the dose of one or more of my care recipient’s medicines** | **5 (10.6)** | **24 (82.8)** | **0.025** | **<0.001***** | **0.022** | **<0.001***** |
| 4 | I think one or more of my care recipient’s medicines may not be working | 11 (22.4) | 18 (66.7) | 0.145 | <0.001*** |  |  |
| 5 | I believe one or more of my care recipient’s medicines may be currently giving them side effects | 11 (24.4) | 18 (58.1) | 0.234 | 0.004** |  |  |
| *Concerns about stopping* | | | | | | | |
| 1 | I would be reluctant to stop one of my care recipient’s medicines that they had been taking for a long time | 24 (44.4) | 5 (22.7) | 2.720 | 0.083 |  |  |
| 2 | I get stressed whenever changes are made to my care recipient’s medicines | 13 (40.6) | 16 (36.4) | 1.197 | 0.706 |  |  |
| 3 | I feel that if I agreed to stopping one of my care recipient’s medicines then this is giving up on them | 14 (43.8) | 15 (34.1) | 1.504 | 0.393 |  |  |
| 4 | The person that I care for has had a bad experience when stopping a medicine before | 2 (18.2) | 27 (41.5) | 0.313 | 0.157 |  |  |
| *Involvement* | | | | | | | |
| 1 | I know exactly what medicines the person that I care for is currently taking and/or I have an up-to-date list of their medicines | 25 (41.7) | 3 (25.0) | 2.143 | 0.287 |  |  |
| 2 | I like to know as much as possible about my care recipient’s medicines | 27 (39.1) | 1 (33.3) | 1.286 | 0.841 |  |  |
| 3 | I like to be involved in making decisions about my care recipients medicines with their doctors | 24 (40.0) | 4 (33.3) | 1.333 | 0.666 |  |  |
| 4 | I always ask the doctor, pharmacist or other healthcare professional if there is something I don’t understand about my care recipient’s medicines | 26 (42.6) | 2 (18.2) | 3.343 | 0.143 |  |  |

Concerns factor reversed (no=1, yes=0)

Outcome reversed (no=1, yes=0)
